# Targeted ablation of epicardial ganglionated plexi during cardiac surgery with pulsed field electroporation (*NEURAL AF*)

**DOI:** 10.1007/s10840-023-01615-8

**Published:** 2023-08-10

**Authors:** Daniel R. Musikantow, Vivek Y. Reddy, Ivo Skalsky, Tamaz Shaburishvili, Martin van Zyl, Barry O’Brien, Ken Coffey, John Reilly, Petr Neuzil, Samuel Asirvatham, Joris R. de Groot

**Affiliations:** 1 Helmsley Electrophysiology Center, Icahn School of Medicine at Mount Sinai, Box 1030, One Gustave L. Levy Place, New York, NY 10029 USA; 2 Homolka Hospital, Prague, Czech Republic; 3 Tbilisi Heart and Vascular Clinic, Tbilisi, Georgia; 4 Royal Jubilee Hospital, Victoria, BC Canada; 5 AtriAN Medical, Galway, Ireland; 6 Mayo Clinic, Rochester, MN USA; 7 University Medical Center, Amsterdam, The Netherlands

**Keywords:** Cardiac autonomic nervous system, Atrial fibrillation, Pulsed field ablation, Post-operative atrial fibrillation

## Abstract

**Background:**

Modulation of the cardiac autonomic nervous system (ANS) is a promising adjuvant therapy in the treatment of atrial fibrillation (AF). In pre-clinical models, pulsed field (PF) energy has the advantage of selectively ablating the epicardial ganglionated plexi (GP) that govern the ANS. This study aims to demonstrate the feasibility and safety of epicardial ablation of the GPs with PF during cardiac surgery with a primary efficacy outcome of prolongation of the atrial effective refractory period (AERP).

**Methods:**

In a single-arm, prospective analysis, patients with or without a history of AF underwent epicardial GP ablation with PF during coronary artery bypass grafting (CABG). AERP was determined immediately pre- and post- GP ablation to assess cardiac ANS function. Holter monitors were performed to determine rhythm status and heart rate variability (HRV) at baseline and at 1-month post-procedure.

**Results:**

Of 24 patients, 23 (96%) received the full ablation protocol. No device-related adverse effects were noted. GP ablation resulted in a 20.7 ± 19.9% extension in AERP (*P* < 0.001). Post-operative AF was observed in 7 (29%) patients. Holter monitoring demonstrated an increase in mean heart rate (74.0 ± 8.7 *vs.* 80.6 ± 12.3, *P* = 0.01). There were no significant changes in HRV. There were no study-related complications.

**Conclusions:**

This study demonstrates the safety and feasibility of epicardial ablation of the GP using PF to modulate the ANS during cardiac surgery. Large, randomized analyses are necessary to determine whether epicardial PF ablation can offer a meaningful impact on the cardiac ANS and reduce AF.

**Trial registration:**

Clinical trial registration: NCT04775264.

**Supplementary Information:**

The online version contains supplementary material available at 10.1007/s10840-023-01615-8.

## Introduction

Catheter ablation of atrial fibrillation (AF) by circumferential isolation of the pulmonary veins has been demonstrated to yield a 70–80% freedom from symptomatic atrial arrhythmias out to one year in patients with paroxysmal AF [1, 2]. It has been posited, however, that the effects of pulmonary vein isolation (PVI) are derived not only from treatment of the ectopic foci triggering AF but also from modulation of the cardiac autonomic nervous system (ANS) by coincident or intentional ablation of the cardiac ganglionated plexi (GP) surrounding the left atria.

This suggestion is supported by the fact that PVI performed using either radiofrequency (RF) or cryothermal energy frequently results in an immediate vagal response during ablation and in an increase in the resting heart rate during follow-up, both of which are associated with freedom from recurrent arrhythmias [3]. Indeed, parasympathetic stimulation has been demonstrated to shorten the atrial effective refractory period (AERP) in a heterogeneous manner which, in turn, has been demonstrated to provide the substrate for initiation and maintenance of atrial fibrillation [4–8]. Accordingly, patients with a history of AF have been shown to have a shorter AERP when compared to similar controls [9].

As a result, the cardiac ANS remains an attractive target for the treatment of atrial arrhythmias. However, trials modulating the ANS in the treatment of AF have shown conflicting results. A large randomized trial in paroxysmal AF demonstrated better outcomes with PVI + GP ablation with RF than either treatment alone, an effect which is difficult to distinguish from that achieved through improved PVI from additional ablation around the pulmonary veins [10, 11]. Conversely, the *AFACT* trial, in which randomized patients have AF to epicardial GP ablation with RF in addition to PVI and left atrial lines, showed neutral outcomes with regard to AF recurrence [12, 13]. One of the hypotheses underlying these results is that GP ablation with RF may be associated with atrial myocardial ablation, which, in itself, can create a new arrhythmogenic substrate.

Pulsed field (PF) ablation is a predominately non-thermal ablative technology which has been gaining momentum in the field for cardiac ablation in part because of its relative preferentiality to ablation of cardiac myocytes—allowing for PVI while largely sparing many nearby structures such as the esophagus and phrenic nerve [14, 15]. Recent clinical evidence suggests that PVI using PF energy results in a highly attenuated effect on the ANS when compared with conventional thermal ablation–radiofrequency or cryoablation [16, 17]. On the other hand, recent in vitro evidence demonstrates the ability for PF to affect a neuron cell model at dosages comparable to that seen with cardiac myocytes [18]. This discrepancy may be at least in part explained by both the distance of the GP from an endocardial source of pulsed field energy and the potential reduction in PF energy by the insulating effects of epicardial fat [19].

Therefore, to optimally target the GPs with PF and avoid myocardial ablation, an epicardial approach may be needed. Indeed, employing a surgical epicardial approach in an animal model using high-dose PF energy has demonstrated relatively selective efficacy in GP ablation when compared to radiofrequency energy, with less damage to surrounding myocytes [20, 21]. Herein, in the proof of principle *NEURAL AF* study, we assessed the feasibility of using direct application of PF energy using an epicardial approach in patients undergoing coronary artery bypass grafting (CABG) to modulate the ANS and increase atrial refractoriness.

## Methods

### Study design

*NEURAL AF* (NCT04775264) is a multi-center, single-armed, prospective clinical study to assess the safety and feasibility of using PF energy targeted at the ganglionated plexi in patients undergoing open-chest CABG surgery using a proprietary open chest epicardial deganglionation system (OCED; AtriAN Medical Ltd., Galway, Ireland). Patients were included regardless of prior history of atrial fibrillation, but were excluded if they had any prior history of PVI (see Table S1 for full list of inclusion/exclusion criteria). The primary efficacy endpoint of the study was a change in atrial autonomic tone as demonstrated by AERP prolongation tested intra-procedurally. Secondary endpoints included changes in heart rate and heart rate variability (HRV), including standard accepted metrics such as standard deviation of the normal sinus beats (SDNN), number of pairs of successive normal-beat intervals differing more than 50 ms (NN50), and spectral analysis, through pre- and post-procedure Holter monitors. Additionally, the incidence of post-operative AF (POAF), defined as any AF occurrence lasting longer than 30 s, was examined during the course of the index hospital admission. POAF was monitored through continuous ECG monitoring at the ICU or clinical ward.

The study was approved by the Czech National Competent Authority SUKL and the local ethic committees and was performed in full accordance with the principle of the “Declaration of Helsinki” (2013). All patients underwent informed consent in advance of treatment. All events were reviewed by an independent Clinical Events Committee (CEC).

### Ablation system

**Fig. 1:**
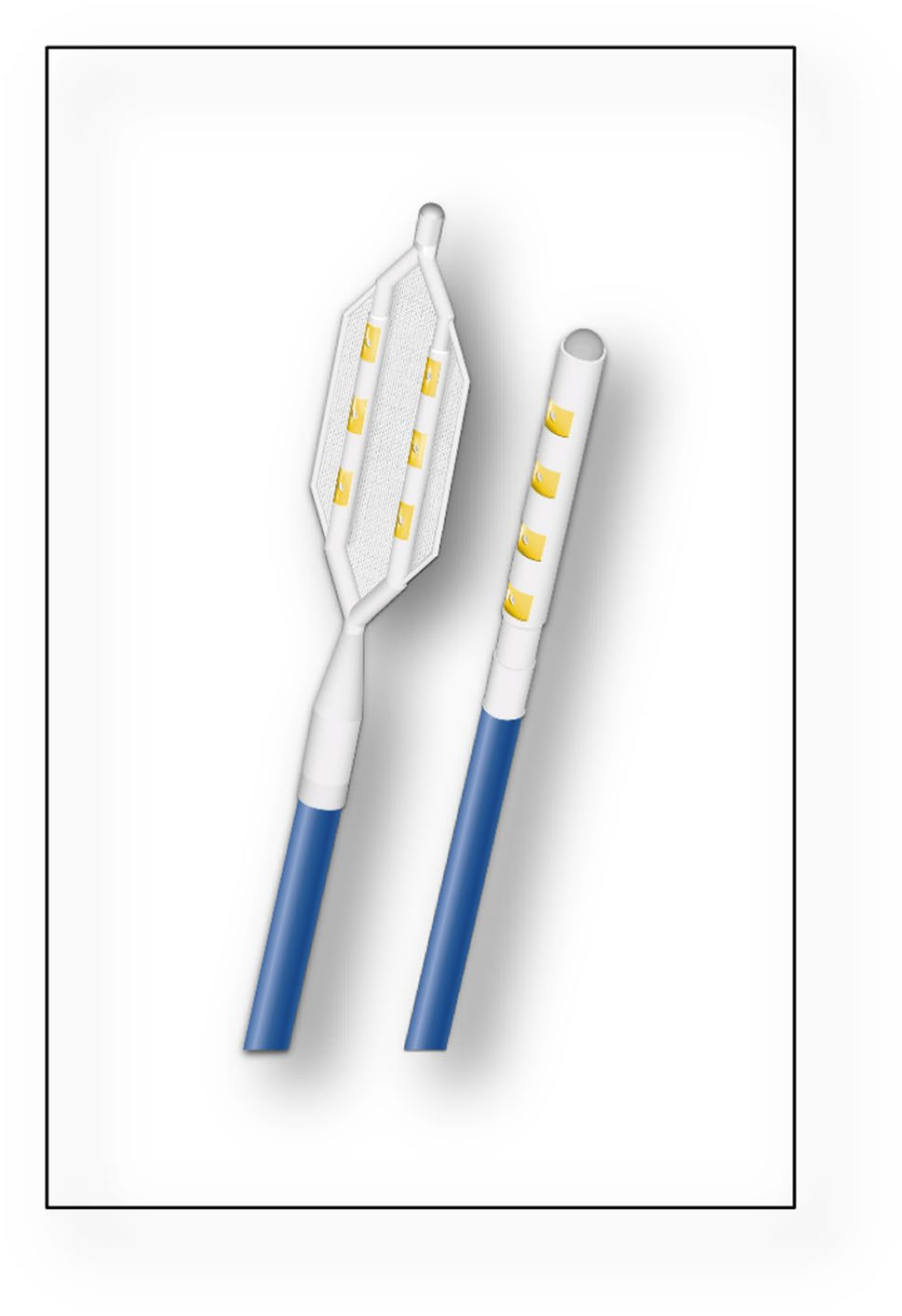
Catheter configurations available for epicardial pulsed field ablation. Schematic of the two available catheters used in this study. Left: “Glove” catheter used for ablation within the oblique sinus. Right: “Finger” catheter used for the remaining GPs

Epicardial GP ablation was carried out using the OCED system, which consists of two catheters as well as a compatible PF generator (Fig. 1). The catheters were available with either a “finger” configuration consisting of four electrodes on a single spline or a “glove catheter” which includes six electrodes divided across two splines. Each electrode was irrigated with saline and insulated to direct the fields towards the epicardial surface. The catheters were attached to a PF generator which provided 1000 V DC pulses of 100 µs duration.

### AERP measurement

A standard diagnostic EP catheter was positioned at the back of the atrium for recording atrial activation during pacing. AERP was then assessed by delivering single extra-stimuli at a drive train of 500 ms from a temporary epicardial wire placed distant from the region of ablation at either the left atrial appendage or right atrium. AERP was defined as the longest extra-stimulus interval which does not capture atrial tissue. In the event that pacing was performed from two separate sites, the combined left and right atrial average was used for comparison. Baseline assessments were performed after administration of paralytic agents and, in some cases, after cannulation for bypass. Repeat measurements were conducted at the conclusion of ablation.

### Ablation procedures

**Fig. 2:**
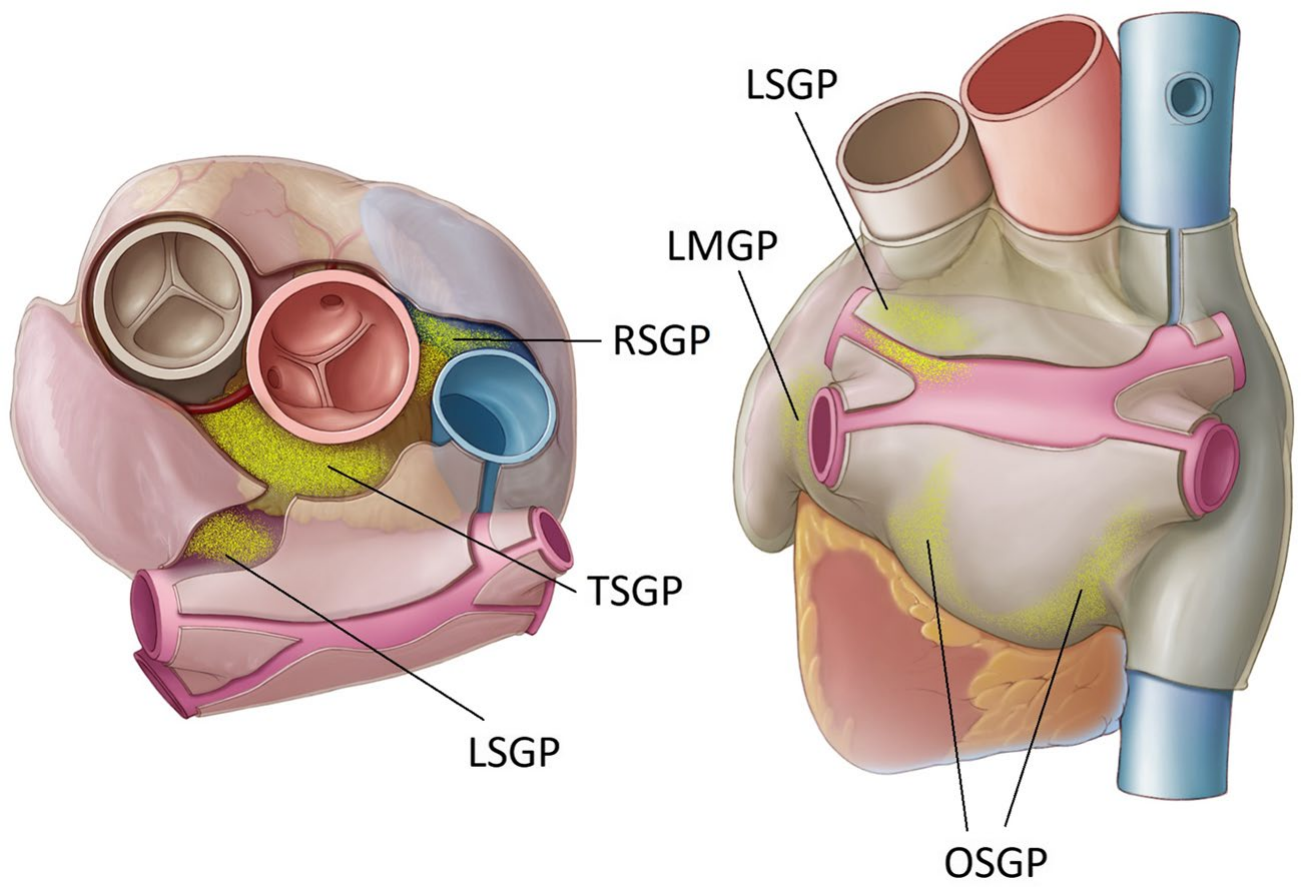
Locations of the targeted ganglionated plexi. Schematic demonstrating locations of the GP. OSGP: oblique sinus GP located on the posterior left atrium near the inferior pulmonary veins; RSGP: right superior GP on the anterosuperior surface of the right atrium, medial to the superior vena cava and lateral to the aortic root; TSGP: transverse sinus GP located at the base of the aorta and pulmonary artery within the transverse sinus; LSGP: left superior GP near the junction of the left superior pulmonary vein and left atrial roof; LMGP: ligament of Marshall GP which runs along the ligament of Marshall between the left-sided pulmonary vein and left atrial appendage

**Fig. 3:**
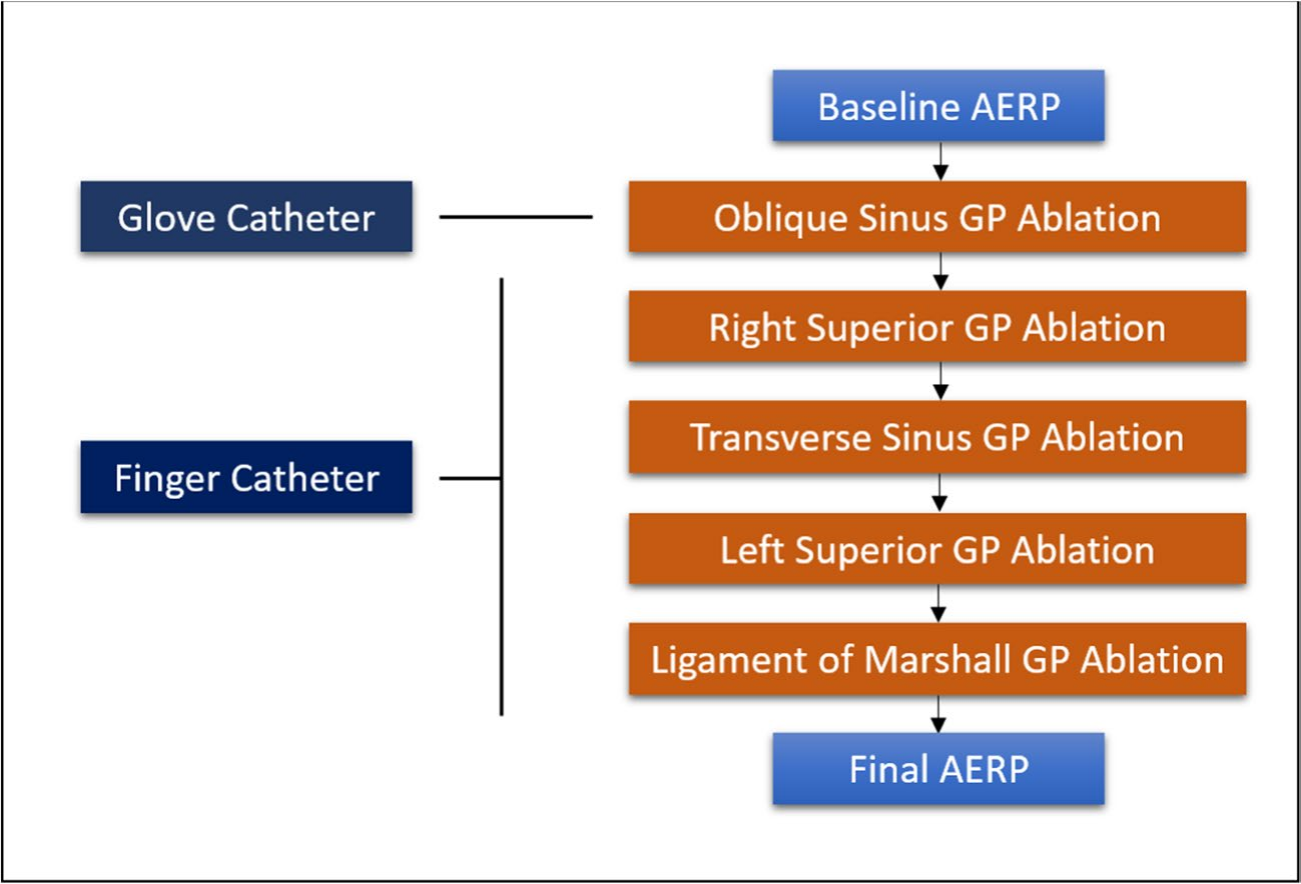
Prescribed sequence of ablation and AERP measurements performed

Procedures were performed at two sites (Tbilisi Heart & Vascular Clinic, Tbilisi, Georgia & Na Homolce Hospital, Prague, Czech Republic). Patient preparation, anesthesia, and sternotomy were performed as part of standard operating procedure. In addition to general anesthesia, a paralytic agent (vecuronium or rocuronium) was administered to all patients. The epicardial fat pads, in which the GPs reside, were visually identified. Five total GPs were targeted in a prescribed sequence, and the oblique sinus (OSGP) was treated with the glove catheter while the remaining sites were treated with the finger catheter (Figs. 2 and 3). Each ablation sequence consisted of 10 ECG-gated pulses each at 1000 V and 100 µs in duration while irrigating the catheter at 2 ml/min using 0.9% normal saline solution. Up to 6 sequences were delivered to each treatment site. Pull back or re-positioning of the catheter was allowed between pulses to ensure adequate coverage of the target area.

### Holter monitoring

All patients received 24-h Holter monitoring at baseline and one-month post-ablation (Cardiologs, Philips, Amsterdam, Netherlands or Biomedical Instruments, Michigan, United States). Heart rate variability (HRV) was assessed using standard accepted metrics such as standard deviation of the normal sinus beats (SDNN), number of pair of successive normal-beat intervals differing more than 50 ms (NN50), and spectral analysis.

### Statistical design

This study was powered to detect a relative increase in AERP of 10%. Assuming a mean AERP of 250 ms with a 12.5-ms standard deviation, a sample size of 21 patients would have a 95% power of detecting this difference with an alpha of 0.1%. All values are reported as mean ± standard deviation. Comparative analysis was performed using a paired-*t* test, Student’s *t*-test, or Fisher’s exact test where appropriate. All analyses were performed using SPSS V 24 (IBM Corp., Armonk, NY).

## Results

### Subject demographics

A total of 25 patients were screened for the procedure, 24 of whom met the inclusion/exclusion criteria, signed consent, and underwent the study protocol. Of the 24 patients, 3 (13%) had a pre-operative history of atrial fibrillation. The majority (80%) of patients were receiving beta-blockers leading up to the procedure, which were continued leading up to the surgery. The remainder of the baseline characteristics are found in Table 1.

**Table 1:** Baseline demographics of all patients undergoing experimental protocol

Baseline values (*N* = 24)	Mean ± ST Dev
Age (years)	61.1 ± 7.7
BMI (kg/m^2^)	29.4 ± 4.4
LA diameter (cm)	3.7 ± 0.4
LVEF (%)	49.7 ± 7.4
Baseline demographics	*N* (%)
Gender (male)	17 (71)
Paroxysmal AF	3 (13)
Coronary artery disease	24 (100)
Prior MI	2 (8)
Hypertension	23 (96)
Diabetes	9 (38)
Stroke or TIA	0
β-blockers	19 (79)
Class I or III anti-arrhythmic	0

*BMI* body mass index, *LA* left atrium, *LVEF* left ventricular ejection fraction, *MI* myocardial infarct, *TIA* transient ischemic attack

### Ablation procedure

Access to all of the targeted ablation sites as well as delivery of PF energy was successful in all 24 patients. One patient experienced hypotension during PF delivery and proceeded immediately to revascularization before all of the PF sequences were delivered (only OSGP, RSGP, and TSGP were treated). This was attributed to a nearly occluded left anterior descending artery (LAD). In all, 3 serious adverse events (SAE) and 8 adverse events (AE) were identified, none of which were attributed by the CEC to the epicardial ablation procedure (Table S2).

### Impact on atrial refractoriness

**Fig. 4:**
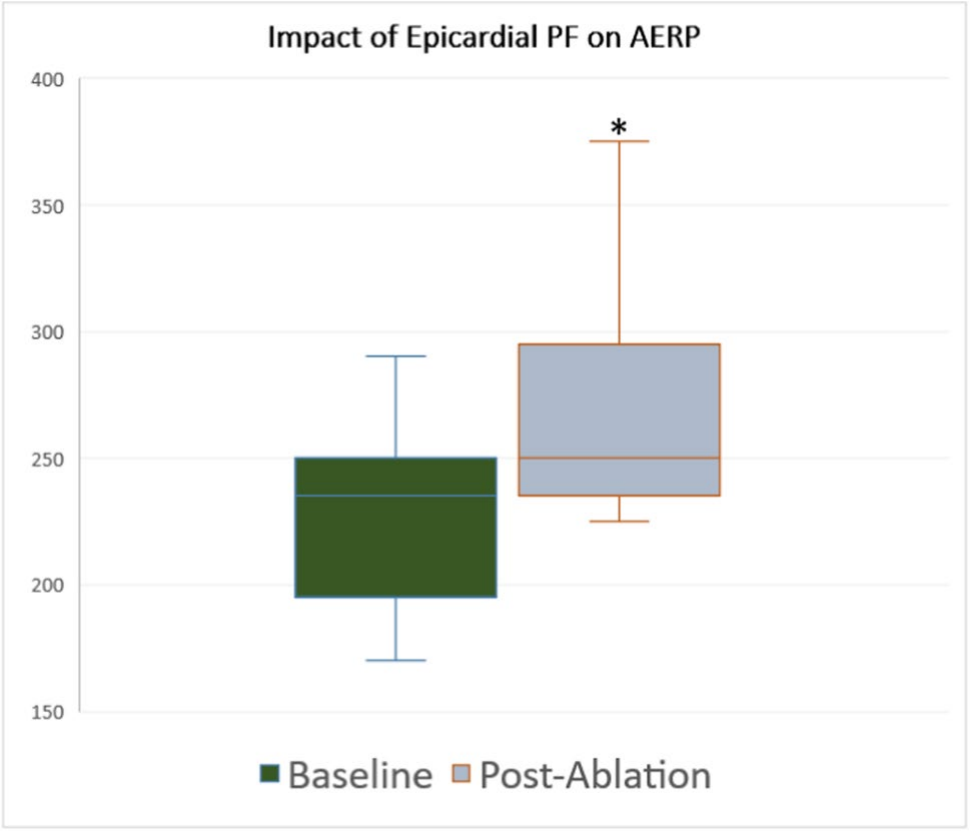
Impact of PF on AERP. Change in AERP between baseline (226 ± 45 ms), and post-GP ablation (269 ± 57 ms). * denotes *P* < 0.05

Pre-ablation and corresponding post-ablation AERP values were available for 17 (71%) of patients. For the remaining 7 patients, there were technical issues with the AERP measurement that precluded measuring both pre- and post-procedural AERP. On average, AERP measurements were taken from 1.7 ± 0.47 sites. The baseline AERP was 226 ± 45 ms and increased to 269 ± 57 ms post-PF procedure, representing a 20.7 ± 19.9% extension in ERP (*P* < 0.001) (Fig. 4). Including only measurements from the RA, baseline AERP increased from 241 ± 40 to 287 ± 55ms, representing a similar 20.0 ± 17.6% change (*P* < 0.001).

### Post-operative course

All 24 patients were followed for the remainder of their hospitalization on cardiac telemetry and none received any anti-arrhythmic drugs prophylactically. POAF was seen in 7 (29%) of the patients, none of whom had a pre-existing history of atrial fibrillation (Table 2). Treatment of POAF included beta-blockers, amiodarone, and cardioversion when necessary. Patients who developed atrial fibrillation had a trend toward smaller extension of 35 ± 46 ms in AERP with ablation *vs*. 40 ± 45 ms in patients who did not develop POAF (*P* = 0.06).

**Table 2:** Characteristics of post-operative atrial fibrillation. First AF time point is measured as time from ICU admission to AF detection

Patient	Duration of hospital stay	Duration of telemetry monitoring	First AF timepoint	First AF event duration	Longest AF event	AF burden	Treatment
01–001	7 d	6 d 11 h 52 min	2 d	5 h	8 h 42 min	7.8%	
01–002	6 d	5 d 15 h 16 min	1 d 6 h	2 h 46 min	2 h 46 min	2.4%	DCCV
01–011	9 d	8 d 10 h 45 min	15 h	2 h 45 min	6 h 22 min	4.7%	AAD
01–014	7 d	6 d 15 h 7 min	2 d 11 h	2 h	3 h 54 min	3.8%	AAD/DCCV
02–001	10 d	9 d 4 h 27 min	2 d 18 h	15 h 53 min	1 d 3 h	19%	AAD/DCCV
02–004	12 d	11 d 17 h 42 min	1 d 11 h	14 h 59 min	14 h 59 min	7.4%	AAD
02–005	7 d	6 d 9 h 37 min	3 d 21 h	1 h 8 min	1 h 8 min	0.7%	AAD

*DCCV* electrical cardioversion, *AAD* chemical cardioversion

### Holter monitor

Pre- and 1-month post-procedure data was available in 22 (92%) patients—two patients experienced technical difficulties or were lost to follow-up. There was a significant increase in both the average and minimum heart rates (74.0 ± 8.7 *vs*. 80.6 ± 12.3 BPM, *P* = 0.01 and 57.0 ± 8.8 *vs*. 61.7 ± 10.2 BPM; *P* < 0.01 for average and minimum, respectively). In terms of heart rate variability, there were some trends in decrease in SDNN (188 ± 31 Pre vs 104 ± 40 1 Mo, *P* = 0.20), but no significant overall differences were seen between baseline and 1-month follow-up (Table 3). Use of beta-blockers was increased post-operatively (79% *vs.* 100%, *P* = 0.05), and amiodarone was initiated in one patient.

**Table 3:** Holter heart rate and heart rate variability analysis. Results of 24-h Holter monitoring comparing pre-procedure (baseline) and 1-month post-procedure heart rates and heart rate variability

	Baseline	1 month	*P*-value
MIN HR	57.0 ± 8.8	61.7 ± 10.2	< 0.01
AVERAGE HR	74.0 ± 8.7	80.6 ± 12.3	0.01
MAX HR	106 ± 17.2	108.4 ± 16.2	0.65
SDNN	188 ± 32	104 ± 40	0.20
SDNN INDEX	58 ± 21	52 ± 37	0.52
RMSSD	62 ± 50	70 ± 70	0.60
NN50	6842 ± 10110	7076 ± 12168	0.91
PNN50	9.4 ± 16.4	7.5 ± 12.3	0.46
LF/HF RATIO	3.0 ± 1.2	2.7 ± 1.8	0.51

*HR* heart rate, *SDNN* standard deviation of the *N–N* intervals, *RMSSD* root mean square of the successive difference, *NN50* number of successive *N*–*N* intervals varying by more than 50 ms, *pNN550* percentage of RR intervals varying by more than 50 ms, *HF* high frequency, *LF* low frequency

*P* values represent the results of paired *t*-tests

## Discussion

This is a first-in-human analysis employing targeted ablation of the epicardial ganglionated plexi using PF electroporation with the intention of modulating the cardiac autonomic nervous system. This was accomplished using a proprietary catheter system via a direct epicardial approach prior to cardiac bypass surgery. The complete course of pulsed field energy was successfully delivered in 23/24 (96%) of patients with one patient undergoing expedited revascularization due to instability unrelated to the study procedure; no procedural related adverse events were noted.

The primary efficacy endpoint was met, as AERP increased by 20.7 ± 19.9% (*P* < 0.001) after the GP ablation with pulsed field energy. While the standard deviation of the effect size is large, this may be explained by the fact that sequential AERP data was only available in 17 (71%) of patients in an already small cohort. POAF was seen in 7 (29%) of patients—which is a comparable rate to a historical control of patients undergoing bypass surgery [22].

Holter monitoring was available in the majority (92%) of patients. While this study was not powered for detecting differences in heart rates or HRV pre- and post-ablation, differences were noted in mean and minimum heart rates as well as trends towards differences in certain HRV metrics such as SDNN and pNN50, consistent with modulation of the ANS. It is important to highlight, however, that CABG itself has been associated with similar findings which is in-part why intra-procedural AERP was chosen as the primary endpoint of this analysis [23, 24].

While small, this study has several implications for the treatment of atrial fibrillation. It is the first such analysis to demonstrate an effect of pulsed field electroporation on the cardiac autonomic nervous system. One of the potential limitations of PF in the percutaneous catheter-based treatment of AF is its sparing of the epicardial ganglionated plexi during routine PVI. This may be due to the large distance between the GPs and the endocardium combined with the insulatory effects of the epicardial fat surrounding the GPs. The ability of direct epicardial delivery of PF energy to impact the ANS paves the way for additional research into configuring waveforms that target neuronal tissue while sparing the myocardium and dosing to have a similar effect from an endocardial approach. Alternatively, the existing technology may be reconfigured to deliver this treatment in a minimally invasive manner, such as via a percutaneous sub-xiphoid approach, to offer ANS modulation outside of the cardiac surgical setting. However, the need and use of ANS modulation in the setting of AF treatment still has to be proven in larger clinical trials.

From a practical standpoint, POAF after cardiac surgery is a common finding with an incidence of ~ 30% [22, 25]. It is associated with an increase in thromboembolic events, recurrent AF, and 1-year mortality [26]. Accordingly, several attempts have been made at reducing the incidence of POAF including prophylactic use of anti-arrhythmic drugs, namely, amiodarone, as well as therapies targeted directly at the ANS [27, 28]. Botulinum toxin, which prevents the neuronal release of acetylcholine and hence has anti-cholinergic effects, has been injected directly into epicardial fat pads. This has been met with mixed results with one randomized 60-person trial demonstrating a 23% absolute risk reduction in POAF in patients with a history of paroxysmal AF [29]. This effect proved durable with an 81% relative reduction in the burden of AF at three years [30]. A second study randomized 130 patients without a history of AF undergoing cardiac surgery and was met with a non-significant 11% absolute risk reduction in atrial arrhythmias [31]. A similar approach using CaCl_2_**,** which induces calcium-induced cell death, was injected into 200 patients undergoing CABG surgery and demonstrated a 21% absolute risk reduction in POAF [32].

In contrast to these agents, PF ablation of the epicardial GPs may offer enhanced safety with fewer off-target effects. In a canine model, electroporation resulted in complete loss of GP cellularity in histological analysis [20]. In contrast to radiofrequency energy, there was no apparent injury to the underlying atrial myocardium. While further in vivo analysis will be needed to confirm the absence of atrial myocardial injury, this may prevent the potential pro-arrhythmic effects noted with thoracoscopic ablation of the GPs with RF. Furthermore, on gross examination, there was no evidence of injury to surrounding tissues including coronary arteries and the esophagus on gross examination. Similar to endocardial PF’s improved safety profile in catheter ablation, epicardial PF offers selective, targeted modulation of the cardiac ANS.

### Limitations

This is a small, single-arm, feasibility and safety analysis on ablation of epicardial GPs using PF energy. The primary endpoint is intraoperative prolongation of AERP, which affords insight into acute changes in the ANS but does not prove durability nor does it examine the potential for anti- or pro-arrhythmic effects of parasympathetic nervous system modulation or atrial or ventricular arrhythmias. Furthermore, as there is no control arm in this analysis, it is not possible to definitively discern the impact of PF on the cardiac ANS from that which naturally occurs normally during the perioperative period. Finally, this study only includes patients undergoing coronary artery bypass surgery; use of this strategy during other cardiac surgeries remains to be demonstrated.

### Conclusions

This study demonstrates the safety and feasibility of using epicardial GP ablation with PF electroporation to modulate the ANS. While interpretation of efficacy is limited by small sample size, an acute prolongation of AERP was noted after GP ablation. Larger, randomized analyses are necessary to determine whether epicardial GP ablation with PF modulates the cardiac ANS in a durable manner and has a meaningful impact on reductions in atrial fibrillation.

## Supplementary Information

Below is the link to the electronic supplementary material.

Supplementary file1 (DOCX 35 KB)

## Data Availability

Data available on request from the authors.

## Abbreviations

AAD: Anti-arrhythmic drugs
AF: Atrial fibrillation
ANS: Autonomic nervous system
GP: Ganglionated plexi
PFA: Pulsed field ablation
PV: Pulmonary veins
PVI: Pulmonary vein isolation
RFA: Radiofrequency ablation
