# Supplementary material for: Targeted ablation of epicardial ganglionated plexi during cardiac surgery with pulsed field electroporation (NEURAL AF)
Source: J Interv Card Electrophysiol. 2023 Aug 10;68(2):467–74. doi: 10.1007/s10840-023-01615-8 (PMC12043773; doi:10.1007/s10840-023-01615-8)
Supplement: Supplementary file 1 — Supplementary file1 (DOCX 35 KB) [file 10840_2023_1615_MOESM1_ESM.docx]

*Online Supplement to:*

**Targeted Pulsed Field Ablation of Epicardial Ganglionated Plexi During Cardiac Surgery (*NEURAL AF*)**

Outline

Page 1: Inclusion/Exclusion Criteria

Page 2: Summary of Adverse Events and Severe Adverse Events

Page 3: Additional Disclosures

**Supplemental Table S1: Inclusion/Exclusion Criteria**

Inclusion criteria:

- - Age is between 18 and 70 years.
  - Scheduled for open-chest cardiothoracic surgery, for coronary artery bypass grafting.
  - Legally competent and willing to sign the informed consent.
  - Life expectancy of at least 2 years.

Exclusion criteria:

- - Previous cardiac surgery.
  - Prior pericardial interventions.
  - Prior pulmonary vein isolation (PVI).
  - Previous or existing pericarditis.
  - Persistent or long-standing persistent atrial fibrillation.
  - Indication for surgical ablation or PVI for atrial fibrillation.
  - Indication for concomitant surgical valve repair or replacement.
  - Indication for concomitant left atrial appendage (LAA) ligation or excision.
  - History of previous radiation therapy on the thorax.
  - History of previous thoracotomy.
  - Prior electrical or mechanical isolation of the Left Atrial Appendage (LAA).
  - The presence of LAA occlusion devices, coronary stents, prosthetic heart valves,

pacemakers or implantable cardioverter defibrillators (ICDs).

- - Myocardial infarction within the previous 2 months.
  - NYHA Class IV heart failure symptoms.
  - Left ventricular ejection fraction (LVEF) < 40%, measured by transthoracic
  - echocardiography (TTE).
  - Left atrial (LA) diameter > 5.0 cm, measured by transthoracic echocardiography (TTE).
  - The presence of left atrial thrombus when examined by transoesophageal

echocardiography (TEE).

- - The presence of AF attributable to non-cardiovascular causes such as thyroid disease,
  - electrolyte imbalance/dehydration or other reversible causes.
  - Active infection or sepsis as evidenced by increased white blood cell count, elevated

CRP or temperature > 38.5°C.

- - Known or documented carotid stenosis > 80%
  - Stroke or transient ischemic attack within the previous 6 months.
  - Known or documented epilepsy.
  - Pregnancy or child-bearing potential without adequate contraception.
  - Circumstances that prevent follow-ups.
  - Drug abuse.
  - Patients cannot be enrolled in another clinical study

**Supplemental Table S2: Summary of Adverse Events and Serious Adverse Events**

| Patient # | Description | Severity | Treatment | Resolved | Relationship to Device | Relationship to Procedure |
| --- | --- | --- | --- | --- | --- | --- |
| Severe Adverse Events | | | | | | |
| 01-011 | Post-operative AF | Mild | Chemical and Electrical Cardioversion | Y | N | Possible |
| 02-001 | Pulmonary Embolism | Severe | Anticoagulation | Y | N | Possible |
| 02-002 | Post-operative AF | Moderate | Chemical and Electrical Cardioversion | Y | N | Possible |
| Adverse Events | | | | | | |
| 01-002 | AF during treatment | Moderate | Chemical Cardioversion | Y | N | Possible |
| 01-002 | AF during CABG procedure | Mild | Electrical Cardioversion | Y | N | Possible |
| 01-002 | Post-Operative AF | Mild | Chemical Cardioversion | Y | N | Possible |
| 01-003 | Hypotension and AF during treatment | Mild | Termination of treatment and progression to CABG | Y | N | Possible |
| 01-011 | Post-operative AF | Moderate | Chemical Cardioversion | Y | N | Possible |
| 01-011 | Post-operative AF | Mild | Chemical Cardioversion | Y | N | Possible |
| 02-004 | Post-operative AF | Moderate | Chemical Cardioversion | Y | N | Possible |
| 02-005 | Post-operative AF | Moderate | Chemical Cardioversion | Y | N | Possible |

**Supplemental Table S2:** Summary of Severe Adverse Events and Adverse Events encountered during the analysis. **Mild**: Awareness of sign, symptom, or event but easily tolerated. **Moderate**: Discomfort enough to cause interference with usual activity and may warrant intervention. **Severe:** Incapacitating iwht inability to do usual activities or significant affects clinical status, and warrants intervention.

## Additional Disclosures:

Unrelated to this manuscript, Vivek Reddy has served as a consultant for and has equity in Ablacon, Acutus Medical, Affera-Medtronic, Apama Medical-Boston Scientific, Anumana, APN Health, Aquaheart, Atacor, Autonomix, Axon Therapies, Backbeat, BioSig, CardiaCare, CardioNXT / AFTx, Circa Scientific, CoRISMA, Corvia Medical, Dinova-Hangzhou DiNovA EP Technology, East End Medical, EPD-Philips, EP Frontiers, Epix Therapeutics-Medtronic, EpiEP, Eximo, Farapulse-Boston Scientific, Field Medical, Focused Therapeutics, HRT, Intershunt, Javelin, Kardium, Keystone Heart, LuxMed, Medlumics, Middlepeak, Neutrace, Nuvera-Biosense Webster, Oracle Health, Restore Medical, Sirona Medical, SoundCath, Valcare; unrelated to this work, has served as a consultant for Abbott, Biosense-Webster, BioTel Heart, Biotronik, Boston Scientific, Cairdac, Cardiofocus, Cardionomic, CoreMap, Fire1, Gore & Associates, Impulse Dynamics, Medtronic, Novartis, Philips, Pulse Biosciences; and has equity in Manual Surgical Sciences, Newpace, Nyra Medical, Surecor, and Vizaramed.
